# Employment status among cancer survivors in a Late Effects Clinic in Denmark

**DOI:** 10.1007/s11764-023-01496-w

**Published:** 2023-11-25

**Authors:** Annette Sicko Skovgaards, Thea Otto Mattsson, Lærke Kjær Tolstrup

**Affiliations:** 1 Faculty of Health Sciences, University of Southern Denmark, Odense, Denmark; 2 Department of Oncology, Odense University Hospital, Odense, Denmark; 3 Department of Clinical Research, University of Southern Denmark, Odense, Denmark

**Keywords:** Cancer survivor, Employment, Late effects, Patient-reported outcomes, Survivorship questionnaire

## Abstract

**Purpose:**

This study aims to investigate changes in employment status among disease-free working-age cancer survivors (CSs) with late effects from diagnosis to their first meeting in the Late Effects Clinic (LEC) and investigate associated patient-reported outcomes of reduced employment status.

**Methods:**

Retrospective analysis of a cohort of CSs followed in a LEC at a single institution from January, 2022, to March, 2023. Working-age CSs with no current evidence of active cancer were included in this study. CSs completed a baseline questionnaire (EORTC QLQ-SURV100) before their initial consultation. Reduced employment status was defined as transition from being in paid work at diagnosis to working fewer hours or not at all at the first visit. Multivariate linear regression analysis was used.

**Results:**

A total of 119 CSs with diverse cancer types with a mean age of 51 years (range 26 to 70) were included in this study. Eighty percent were female. Of 93 CSs in paid work at diagnosis, 66 (71%) have reduced employment status. Reduced employment status was associated with lower role functioning score (*β* = −12.3, *p* = 0.046), higher loss of income score (*β* = 35.1, *p* = 0.001), and lower Global health status score (*β* = − 8.3*, p* = 0.05).

**Conclusions:**

This study shows that the majority of CSs seen in the LEC have reduced employment status. This is associated with impaired quality of life.

**Implications for cancer survivors:**

Identifying and treating late effects early in cancer survivorship are important to secure CSs’ labour market attachment and, thus, their financial and social well-being.

**Supplementary Information:**

The online version contains supplementary material available at 10.1007/s11764-023-01496-w.

## Background

Due to early detection and improved treatment, the number of cancer survivors (CSs) has been increasing [1]. More than 370,000 (6%) Danes live with or have had cancer. About 60% of these people are of working age [2], and up to 50% of CSs experience late effects such as fatigue, cognitive impairment, sleep problems, and pain [3, 4]. These can affect the CS’s ability to return to their job to the same extent as before their diagnosis. Based on our clinical experience, many CSs are eager to return to work but experience that they have to make adjustments at work; they are not able to work the same hours and/or perform the same work tasks as before the diagnosis. Beyond the financial considerations, being part of the workforce is important for CSs’ psychological and social well-being because of its links to personal identity, self-esteem, life purpose, and social relationships [5]. Hence, there is a growing interest in patient-reported outcome (PRO) data to evaluate CSs’ health-related quality of life (HRQOL). HRQOL covers the subjective perceptions of the positive and negative aspects of CSs’ symptoms, including physical, emotional, social, and cognitive functions and, importantly, disease symptoms and side effects of treatment [6]. The European Organisation for Research and Treatment of Cancer (EORTC) Quality of Life Group has developed an HRQOL assessment strategy that captures the full range of issues relevant to disease-free CSs resulting in the EORTC Quality of Life Cancer Survivorship Core Questionnaire (QLQ-SURV100) [7]. As the EORTC QLQ-SURV100 is a newly developed questionnaire, there is no published data yet available.

In January, 2021, the Region of Southern Denmark decided to establish four Late Effects Clinics (LECs). Primo 2022, the LEC of Odense University Hospital, Denmark, opened. The clinic’s purpose is to offer interdisciplinary help to CSs experiencing severe late effects with unmet needs within the existing framework.

Although many studies have investigated employment status and return to work in CSs, to the best of our knowledge, none has investigated the change in employment among CSs with complex late effects with unmet needs [8–10]. de Moor et al. suggest further research among understudied groups, underlining the relevancy of this study [8].

The main aim of this study was to investigate changes in employment status among disease-free working-age CSs with late effects and unmet needs from diagnosis to their first meeting in the LEC between January, 2022, and March, 2023. A secondary aim is to investigate the association between change in employment status and PRO-data (role functioning score, work score, loss of income score, and global health status score) measured by the EORTC QLQ-SURV100.

## Methods

### Study setting

The database containing data from all CSs seen in the LEC of Odense University Hospital, Denmark, between January, 2022, and March, 2023, was reviewed. Disease-free CSs with complex late effects after cancer treatment can be referred by their general practitioner or hospital departments, when beyond active treatment. Hence, they constitute only a small proportion of the population of CSs. CSs were requested to complete the Danish version of the EORTC QLQ-SURV100 at their first consultation in the LEC. The patient reporting is used as a dialogue tool in the clinical encounter. Based on this consultation, further investigations, referral to other relevant departments, or rehabilitation in the municipalities are initiated. The LEC also offers group-based interventions, individual nurse counselling, or psychological therapy. Informed written consent was obtained from all participants before completing the questionnaire.

### Population

The exclusion criteria were (1) being retired at the first visit, (2) the latest evaluation was with relapse or new cancer, or (3) death. One hundred nineteen survivors attending the LEC were considered eligible.

### Main outcome variables

The main outcome was change in employment status from diagnosis to the first visit to the LEC. CSs reported their employment status at their first meeting, and information on their employment status at diagnosis and up to 1 year prior was collected from a chart audit. Employment status was categorised as paid work (full-time job, part-time job, flex job) and unemployed (disability pension, early retirement, and others (job training, jobseeker, sick leave, education, and unemployed without unemployment benefits)). Among CSs in paid work at diagnosis, change in employment status from diagnosis to the first visit was further explored. Change in employment status was dichotomised into reduced employment status (transition to fewer hours or not working at all) and maintained employment status (same employment status as before).

Sociodemographic data, such as gender (female/male), age at the first visit, cohabitation status (living with partner yes/no), children under 18 years (yes/no), and educational attainment (primary education, upper secondary/ vocational education, or higher (vocational)/university education) were obtained by specialists in the LEC at the first visit. Health-related variables, including cancer site, age at diagnosis, years since primary diagnosis, treatment modalities, years since primary treatment, and comorbidity (yes/no), were extracted from the medical records. Late effects were assessed by specialists within the field of late effects after cancer and its treatment at the first visit to the LEC. In a Danish context, late effects are defined as health problems that occur during treatment and become chronic or develop months or years after treatment has ended [11].

### Tools and measures

The EORTC QLQ-SURV100 is based on the reliable and valid EORTC Quality of Life Core Questionnaire (QLQ-C30). The QLQ-SURV100 is a newly developed and partly validated questionnaire for disease-free CSs and is currently undergoing further validation [7]. The QLQ-SURV100 consists of 100 items, including a global health status/quality of life scale, thirteen functional scales, nine symptom scales, one symptom checklist assessing chronic side effects of cancer treatments, and twelve single items. This study focused on the scales and items of role functioning, work, loss of income, and global health status. The questions in the four scales and items are added in Supplementary File 1. The QLQ-SURV100 questions are rated on a scale from 1 (not at all) to 4 (very much). Except for the global health status items, where a scale from 1 (very poor) to 7 (excellent) is used. The time frame of the questions is “during the past week” for role functioning and global health status. For work and loss of income, the time frame is “since the diagnosis and treatment of your cancer.” The scoring approach for the QLQ-SURV100 is identical in principle for the QLQ-C30. The CSs’ responses are transformed into a standard score according to the official EORTC scoring manual. Missing items were handled as outlined in the scoring manual [12]. The score ranges from 0 to 100, with a higher score for a functional scale indicating a high level of functioning, a higher score for the global health status indicating a high quality of life, but a higher score for a symptom scale indicating a higher level of symptomatic problems.

Study data were extracted from the electronic medical records and double entered into the secure REDCap (Research Electronic Data Capture) [13, 14].

### Statistical analysis

Descriptive statistics were used to summarise the sociodemographic and clinical characteristics of the participants. Univariate analyses including chi-square test, two-sample Wilcoxon rank-sum (Mann–Whitney) test, and two-sample *t*-test were performed to determine any differences in the population characteristics and the standard score of the four domains between the two groups maintained and reduced employment status. For the standard score of the four domains, multivariate linear regression models with the independent variables employment status, gender, cohabitation status, educational attainment, and fatigue were used. One-sample *z*-test was used to determine if the PRO-data scores differed from the nominative data from the Danish population and EORTC QLQ-C30 reference values [15, 16]. Differences were considered significant if the *p*-value was equal to or less than 0.05. Statistical analysis of the data was performed using STATA 17.

## Results

### Sociodemographic and clinical characteristics

One hundred sixty-five CSs were seen in the LEC between January, 2022, and March, 2023. Of the 165 CSs, 41 were excluded due to retirement, four due to relapse, and one because of death. The response rate of the QLQ-SURV was 99%. The mean age at first visit was 51 years (range 26 to 70). Sixty-five percent of the CSs had higher (vocational) or university education. The mean age at diagnosis was 46.5 years (range 19 to 65), and the mean time since primary diagnosis was 4.3 years (range 0 to 31). The full sample sociodemographic and clinical characteristics are presented in Table 1. No statistically significant differences in population characteristics were observed.

**Table 1:** Characteristics of 119 cancer survivors (CSs) at working age at first visit from a Late Effects Clinic (LEC) in Denmark, from January, 2022, to March, 2023, by change in employment status

	Total sample	Change in employment status from diagnosis to first visit among CSs in paid work at diagnosis and at working age at first visit		CSs outside the labour force at diagnosis
	*n* = 119	Maintained *n* = 27	Reduced *n* = 66	*n* = 26
Sociodemographic variables				
Gender, *n (%)*				
Female	95 (80)	20 (74)	55 (83)	20 (77)
Male	24 (20)	7 (26)	11 (17)	6 (23)
Age at first visit, *mean (SD)*	51.3 (10.0)	52.7 (8.6)	50.9 (9.8)	51 (11.8)
Living with partner, *n (%)*	83 (70)	23 (85)	48 (73)	12 (46)
Children < 18 years, *n (%)*	49 (41)	12 (44)	29 (44)	8 (31)
Educational attainment, *n (%) (n = 108)*				
Primary education	6 (5)	2 (8)	2 (3)	2 (10)
Upper secondary/ vocational education	32 (30)	6 (23)	20 (32)	6 (32)
Higher (vocational)/ university education	70 (65)	18 (69)	41 (65)	11 (58)
Health-related variables				
Cancer site^1^, *n (%)*				
Breast	63 (53)	14 (52)	36 (55)	13 (50)
Digestive system	14 (12)	3 (11)	8 (12)	3 (12)
Lung	2 (2)	1 (4)	1 (2)	0
Genital (male)	7 (6)	3 (11)	2 (3)	2 (8)
Genital (female)	14 (12)	3 (11)	8 (12)	3 (12)
Head and neck	7 (6)	2 (7)	2 (3)	3 (12)
Haematological	10 (8)	1 (4)	7 (11)	2 (8)
Melanoma	7 (6)	0	4 (6)	3 (12)
Thyroid	4 (3)	2 (7)	2 (3)	0
Age at diagnosis*, mean (SD)*	46.5 (10.2)	47.4 (9.0)	46.4 (9.5)	45.7 (13.1)
Years since primary diagnosis, *mean (SD)*	4.3 (4.7)	4.6 (6.5)	4.0 (3.7)	4.9 (4.8)
Treatment modalities^1^, *n (%)*				
Surgery	96 (81)	21 (78)	57 (86)	18 (69)
Radiotherapy	66 (55)	16 (59)	34 (52)	16 (62)
Chemotherapy	90 (76)	23 (85)	52 (79)	15 (58)
Immunotherapy	5 (4)	0	3 (5)	2 (8)
Targeted treatment	5 (4)	0	4 (6)	1 (4)
Endocrine treatment	40 (34)	11 (41)	24 (36)	5 (19)
Other^2^	22 (18)	8 (30)	11 (17)	3 (12)
Years since primary treatment, *mean (SD)*	3.1 (3.5)	2.5 (3.1)	2.8 (3.0)	4.5 (4.7)
Comorbidity, *n (%)*				
Yes	72 (61)	16 (59)	37 (56)	19 (73)
No	47 (39)	11 (41)	29 (44)	7 (27)
Late effects, *n (%)*				
Anxiety	12 (10)	1 (4)	6 (9)	5 (19)
Depression	10 (8)	2 (7)	5 (8)	3 (12)
Fear of cancer recurrence	52 (44)	11 (41)	29 (44)	12 (46)
Neuropathy	48 (40)	9 (33)	27 (41)	12 (46)
Cardiac and pulmonary dysfunction	5 (4)	0	4 (6)	1 (4)
Hormonal disorder and infertility	3 (3)	1 (4)	2 (3)	0
Cognitive impairment	64 (54)	16 (59)	38 (58)	10 (38)
Osteoporosis	4 (3)	1 (4)	2 (3)	1 (4)
Lymphoedema	22 (18)	4 (15)	13 (20)	5 (19)
Oral and dental problems	15 (13)	2 (7)	7 (11)	6 (23)
Sex and intimacy	38 (32)	10 (37)	19 (29)	9 (35)
Pain	68 (57)	16 (59)	36 (55)	16 (62)
Eating and swallowing problems	8 (7)	2 (7)	4 (6)	2 (8)
Sleep disturbances	54 (45)	9 (33)	32 (49)	13 (50)
Bowel and urinary dysfunction	32 (27)	6 (22)	16 (24)	10 (38)
Fatigue	87 (73)	19 (70)	53 (80)	15 (58)

*N*, number; *SD*, standard deviation

^1^Categories are not mutually exclusive

^2^Other include zoledronic acid, radioactive iodine, bone marrow transplantation, and stem cell transplantation

### Employment status

**Fig. 1:**
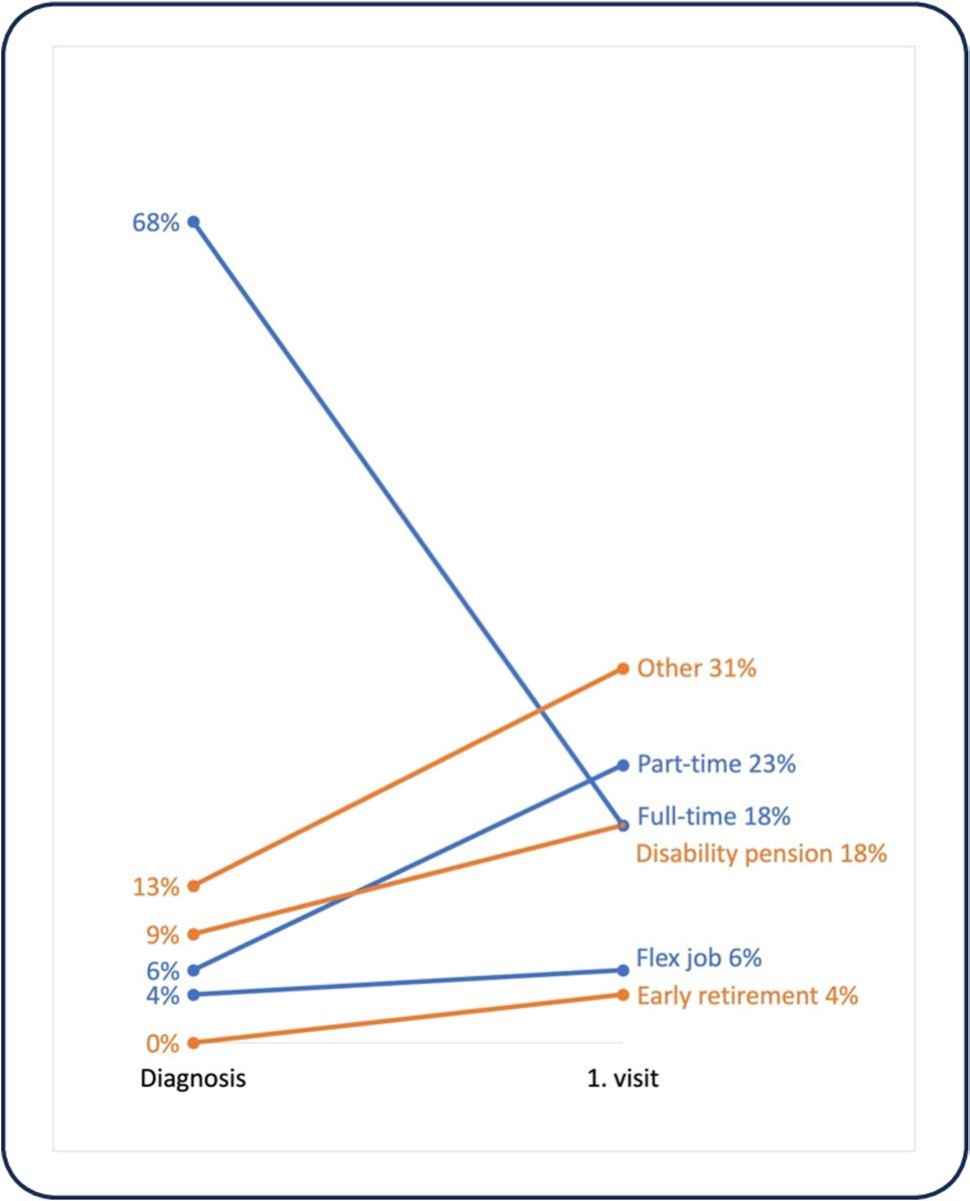
The proportion of employment at diagnosis and first visit among 119 cancer survivors at working age from a Late Effects Clinic in Denmark from January, 2022, to March, 2023. Blue indicates paid work. Orange indicates unemployed. Other include job training, job seeker, sick leave, education, and unemployed without unemployment benefits

Among 119 CSs, 93 (78%) were in paid work at diagnosis and 26 (22%) were unemployed, including 11 (9%) receiving disability pension and 15 (13%) having other job statuses. At the first visit, 56 (47%) were in paid work and 63 (53%) were unemployed, including 21 (18%) receiving disability pension. The proportion of employment status at diagnosis and first visit to the LEC is shown in Fig. 1. Of 93 CSs in paid work at diagnosis, 27 (29%) have maintained employment status and 66 (71%) have reduced employment status. Among the 26 unemployed CSs at diagnosis, four (3%) had a negative change in employment status from other to disability pension. Seven (6%) of the CSs having other job statuses at diagnosis maintained this status. All the CSs receiving disability pension at diagnosis still held disability pension at the first visit. Four (3%) had a positive change in employment status from other to paid work, two got full-time jobs and two got part-time jobs.

### Patient-reported outcomes

In the bivariate analysis, those who had reduced employment status reported a lower role functioning score than CSs with maintained employment status (52.5 vs. 66.7, *p* = 0.012) and a higher loss of income score (55.9 vs. 4, *p* < 0.001) (Table 2). There was no significant difference in the work score or global health status score (Table 2).

**Table 2:** Associations of change in employment and PRO scores of 93 cancer survivors (CSs) at working age and in paid work at first visit from a Late Effects Clinic (LEC) in Denmark, from January, 2022, to March, 2023, by change in employment status—univariate analysis

	Change in employment status from diagnosis to first visit among CSs in paid work at diagnosis and at working age at first visit		*p-value*
	Maintained *n = 27*	Reduced *n = 66*	
EORTC-QLQ-SURV100 (score 0–100)			
Role functioning^1^*, mean (SD) (n = 93)*	66.7 (18.5)	52.5 (28.1)	0.012
Work^1^*, mean (SD) (n = 93)*	70.7 (27.8)	59.2 (29.3)	0.087
Not applicable, *n (n = 10)*	2	8	
Loss of income^2^*, mean (SD) (n = 91)*	4 (11.1)	55.9 (42.6)	<0.001
Not applicable, *n (n = 7)*	1	6	
Global health status^1^*, mean (SD) (n = 91)*	56.5 (17.2)	49.9 (17.4)	0.100

^1^Higher scores indicate better functioning

^2^Higher scores indicate a higher level of problems

In multivariate analysis, controlling for gender, cohabitation status, educational attainment, and fatigue, statistically significant effects between the groups (maintained vs. reduced employment status) were seen for role functioning score (*β* = −12.3, *p* = 0.046), loss of income score (*β* = 35.1, *p* = 0.001), and global health status score (*β* = − 8.3, *p* = 0.05). Fatigue was statistically significantly associated with lower role functioning score (*β* = 14.9, *p* = 0.021) and higher loss of income score (*β* = 21.4, *p* = 0.04). PRO-data associated with changes in employment status in multivariate analyses are shown in Table 3.

**Table 3:** PRO-data of cancer survivors with reduced employment status compared with maintained employment status adjusted for gender, living with partner, educational attainment, and fatigue—multivariate analysis

	RF^1^	*p*	W^1^	*p*	LI^2^	*p*	GHS^1^	*p*
Employment status (ref: maintained employment status)								
Reduced employment status	− 12.3	0.046	− 2.9	0.7	35.1	0.001	− 8.3	0.05
Gender (ref: female)								
Male	7.6	0.3	− 10.2	0.3	− 0.7	0.9	5.5	0.3
Living with partner (ref: yes)								
No	− 6.2	0.3	− 17.5	0.039	16.5	0.1	5.5	0.2
Educational attainment (ref: primary education)								
Upper secondary/vocational education	5.4	0.7	− 9.0	0.6	32.3	0.1	0.6	0.9
Higher (vocational)/university education	− 1.4	0.9	− 26.2	0.1	25.9	0.2	0.9	0.9
Late effect fatigue (ref: no)								
Yes	− 14.9	0.021	− 14.5	0.08	21.4	0.04	− 5.3	0.2

*RF*, role functioning; *W*, work; *LI*, loss of income; *GHS*, global health status

^1^Higher scores indicate better functioning

^2^Higher scores indicate a higher level of problems

Compared to normative data from the general Danish population, the mean role functioning scores and the global health status scores for both those with maintained employment status (*p* < 0.001) and those with reduced employment status (*p* < 0.001) are significantly lower [15]. However, comparing the mean role functioning scores and the global health status scores with EORTC QLQ-C30 reference values, only those with reduced employment status have significantly lower scores (*p* < 0.001) [16].

## Discussion

In this study, the majority of CSs within working age seen in the LEC had reduced employment status from diagnosis to the first visit (71%). Only 29% had maintained employment status. We found that reduced employment status was associated with a lower role functioning score, higher loss of income score, and lower global health status score. There was no significant difference in the work score.

### Employment status

In this study, 78% were in paid work at diagnosis and 9% held disability pension, which corresponds well with the employment rate in the region of Southern Denmark aged 16–64. The general population’s average employment rate was 73% between 2008 and 2021 (range 70 to 77%, highest in 2021), and 7% received disability pension in 2021 [17, 18]. On the first visit to the LEC, 47% were in paid work and 18% held disability pension. Therefore, this study’s findings indicate that CSs with late effects and unmet needs have a higher risk of unemployment and disability pension than the general population.

A systematic review of 28 studies reported data about employment or return to work rates in cancer survivorship. Overall, on average, 63.5% of the participants (range 24–94%) managed to return to work depending on the period after cancer treatment [19].

Although these findings are not directly comparable due to different populations and healthcare systems, they all show that the majority of CSs maintain their employment status after their diagnosis and treatment. This is not in alignment with our findings. However, it may be argued that CSs referred to the LEC may be more affected by the number and complexity of their late effects than the average CS.

### Patient-reported outcomes

In this study, the mean role functioning score and global health status score for those with maintained employment status and for those with reduced employment status were significantly lower than in the general Danish population [15]. This indicates that the CSs seen in the LEC, regardless of employment status, perform worse and have a lower quality of life than individuals without a history of cancer. This is in line with findings from other studies [20, 21]. Compared with EORTC QLQ-C30 reference values for cancer patients, the scores found in this study are significantly lower than the mean role functioning score and global health status score for the CSs with reduced employment, but not for those with maintained employment [16], indicating that CSs with maintained employment status are more like the average cancer patient. However, reduced employment is correlated to limited role functioning and lower quality of life. This is consistent with the findings from a Dutch study, where CSs employed 5 years post-diagnosis had better quality-of-life outcomes compared to those not being employed [9]. Furthermore, in multivariate analysis, we found a significantly lower role functioning score for those with reduced employment status. This suggests that limited role functioning affects the CSs’ ability to return to their job to the same extent as before their diagnosis. This association is in accordance with a Danish study that found that the negative effect of cancer on employment is stronger if the pre-cancer occupation requires high levels of manual skills [22]. In this study, CSs are to a large extent affected by psychological late effects such as fatigue, cognitive impairment, and fear of recurrence. We found that fatigue was statistically significantly associated with lower role functioning score. This is consistent with prior findings [10, 21]. Furthermore, a large proportion of the CSs from the clinic are highly educated. This suggests that there may be a mismatch between individual capabilities and skill demands in the occupation after cancer, which could lead to unemployment. This could be due to late effects. Many CSs are not ready to join the labour market after treatment completion, and the pressure to do so may sustain or worsen the late effects and their ability to work. However, for others, work can have important psychosocial benefits by providing a sense of purpose, creating social connections, and a potential distraction from cancer-related concerns and thereby better management of their late effects.

To fully understand the association between late effects and reduced employment, further research is recommended to collect PRO-data and data on employment status at baseline and regularly with a long follow-up period. Furthermore, prospective studies are needed to assess whether reducing symptoms and improving functioning can improve the workability of CSs. More research is needed to investigate the follow-up data from the LEC.

The findings of this study suggest the need for policies promoting opportunities for more flexible adjustment to jobs where the requirements match the CSs’ abilities. Finally, an unresolved job situation may unnecessarily increase the CSs’ mental workload and cause economic instability.

In this study, CSs with reduced employment status reported a significantly higher loss of income score than CSs who maintained employment. Even though this is not surprising, the result demonstrates the importance of helping survivors back in the labour market, as loss of income has a negative impact on both an individual and societal level. Even in countries with universal health care, there can be additional patient-covered costs of appointments with specialists, medicines, and medical devices [23]. Reduced employment status and loss of income reduce the financial reserves required to meet the direct and indirect medical costs. In addition, CSs may reduce spending on these costs to limit the financial burden, which could result in poorer outcomes regarding late effects, thereby reducing workability.

There is no significant difference in the work score, which could be due to a lack of statistical power in the study.

There is no normative data for the work and loss of income scores, as they were not part of the EORTC QLQ-C30. As the EORTC QLQ-SURV100 is a newly developed questionnaire, there is no published data yet available. We expect more data to be published in the future, both nationally and internationally.

### Strengths and limitations

This study is unique as it is the first from the novel LEC in the region of Southern Denmark. A major strength of this study is the use of real-world data including PRO instruments specifically developed for disease-free CSs, in combination with data from medical records. The medical records for each patient were thoroughly assessed and data was double entered. Furthermore, we had a response rate of 99%, which is extremely high. The high response rate may be due to the fact that the questionnaire was used as a dialogue tool in the clinic, which was motivational for the CSs. Moreover, the questionnaire was completed in connection with the first visit. Accordingly, clinicians were able to encourage completion. All eligible CSs accepted to participate in the study. Another strength of this study is the population of CSs with complex late effects with unmet needs, as employment status has not yet been investigated among this subgroup of CSs. However, this can also be considered a limitation, as the results are not representative of all CSs.

The small sample size is also a limitation. Hence, further studies with larger samples are needed to confirm our findings. Moreover, 80% of the CSs were female, and the results may not be generalisable to men. Previous studies show that women are more likely to seek healthcare, which could explain our results [24, 25]. In this study, over 50% of the included study participants were female breast cancer survivors. Yet, it is estimated in 2021 that 21% of CSs between 15 and 74 years have a history of breast cancer [2]. This demonstrates that this study’s makeup of cancer types is inconsistent with those in long-term cancer survivorship. In this study, 65% of the CSs had a higher education. In addition to gender differences, there is also reported educational differences in the use of health services, indicating social inequality [26, 27]. Compared to CSs with higher education, a greater proportion of CSs with short education are affected by high levels of symptoms and impaired functioning [28]. Therefore, we believe our results may apply to CSs in all educational groups and it underlines the need for this patient group to be referred to a LEC.

The results of this study reflect the Danish welfare model and the unique structures of the health care system, employment law, and culture. As Denmark has a well-developed, tax-financed welfare system, all Danish citizens have equal access to health care and are entitled to limited compensation for loss of income due to unemployment, disability, or illness. Hence, our results may not be generalisable to countries with different welfare systems.

### Implications for clinical practice

In the consultation with the survivors in the clinic, clinicians routinely discuss survivors’ employment status and their ability to work. They explore the physical and cognitive effects of cancer or its treatment, which may limit the survivor’s ability to work. If needed, the LEC assisted by the oncology social workers collaborate with the municipalities to create an individually adapted plan for returning to work at an appropriate pace.

Traditionally, the workplace and the municipal job centre are key actors in helping CSs return to work in Denmark. The role of the job centre is to assist job seekers in their job search, including aiming to reduce reimbursement costs when possible. People who receive unemployment benefits must attend the job centre, and cancer patients may feel pressured to return to the labour market before they are ready [29]. Thus, there is a need for programs tailored to CSs’ circumstances. If the state of health and course of treatment are not considered in cross-sector collaboration, the employment effort may become counterproductive. Therefore, healthcare professionals must identify the CSs’ needs through systematic assessment early in cancer survivorship care, and if possible help remedy late effects or refer the CSs to specialists who can inform them about the existing challenges, opportunities, and offers [4]. Additionally, clinicians should encourage discussion with CSs and their employer to understand workplace options and modifications to their previous role potentially with the help of social workers [4]. Moreover, there is a need to increase referrals for males and CSs with lower education, reducing gender and social inequality. To address this inequality, there is a need to identify CSs with unmet needs, especially among those with few health skills and few resources, through systematic screening of impaired functioning during cancer follow-up [28].

Knowledge of the clinic’s existence must be spread among referring clinicians and general practitioners through more information from the oncology departments and LECs and strengthened cross-sector cooperation.

## Conclusion

This study is the first to present EORTC QLQ-SURV100 data on employment status among CSs with late effects and unmet needs. Three-quarters of CSs within working age seen in the LEC have reduced employment status. This is associated with a lower role functioning score, higher loss of income score, and lower global health status score. Identifying and treating late effects early in cancer survivorship are important to secure CSs’ attachment to the labour market, ensuring their financial and social well-being and reducing the societal socioeconomic burden.

## Supplementary information

ESM 1 (DOCX 17 kb)

## Data Availability

The datasets generated during and/or analysed during the current study are available from the corresponding author on reasonable request. The QLQ-SURV100 can be requested on the website of the EORTC QLG: EORTC Quality of Life Website https://qol.eortc.org/.
