# Supplementary material for: Employment status among cancer survivors in a Late Effects Clinic in Denmark
Source: J Cancer Surviv. 2023 Nov 25;19(2):633–41. doi: 10.1007/s11764-023-01496-w (PMC11925974; doi:10.1007/s11764-023-01496-w)
Supplement: Supplementary file 1 — (DOCX 17 kb) [file 11764_2023_1496_MOESM1_ESM.docx]

# Employment status among Cancer Survivors in a Late Effects Clinic in Denmark

Journal of Cancer Survivorship

Annette Sicko Skovgaards^1,2^, Thea Otto Mattsson^2^, Lærke Kjær Tolstrup^2,3^

1: Faculty of Health Sciences, University of Southern Denmark, Odense, Denmark

2: Department of Oncology, Odense University Hospital, Odense, Denmark

3: Department of Clinical Research, University of Southern Denmark, Odense, Denmark

**Corresponding author**

Annette Sicko Skovgaards

[anhan17@student.sdu.dk](mailto:anhan17@student.sdu.dk)

## Supplementary File 1

The questions in the four scales and items we focused on in the study “Employment status among Cancer Survivors in a Late Effects Clinic in Denmark”: Role functioning, Work, Loss of income, and Global health status from the EORTC QLQ-SURV100.

**Role functioning**

Q8: Were you limited in doing either your work or other daily activities?

Q9: Were you limited in pursuing your hobbies or other leisure time activities?

Q50: Have you been limited in doing physically demanding recreational activities (e.g. swimming or cycling)?

**Work**

Q69: Have your career/job opportunities been limited?

Q70: Do you work fewer hours than you would like?

Q71: Do you perform your work less well?

Q72: Do you lack support and understanding from colleagues and or/management?

**Loss of income**

Q73: Have you lost income?

**Global health status**

Q99: How would you rate your overall quality of life during the past week?

Q100: How would you rate your overall health during the past week?
